# Fe_3_O_4_ magnetic nanoparticle-enhanced radiotherapy for lung adenocarcinoma via delivery of siBIRC5 and AS-ODN

**DOI:** 10.1186/s12967-021-02971-7

**Published:** 2021-08-09

**Authors:** Shuzhen Chen, Fushi Han, Dongdong Huang, Jinqian Meng, Jiapeng Chu, Meng Wang, Peijun Wang

**Affiliations:** 1 Department of Nuclear Medicine, Tongji Hospital, Tongji University School of Medicine, Shanghai, 200065 P.R. China; 2 Department of Emergency Medicine, Shanghai Pulmonary Hospital, Tongji University School of Medicine, Shanghai, 200433 P.R. China; 3 Department of Radiology, Tongji Hospital, Tongji University School of Medicine, Shanghai, 200065 P.R. China; 4 Department of Cardiology, Tongji Hospital, Tongji University School of Medicine, Shanghai, 200065 P.R. China; 5 Department of Radiotherapy, Tongji Hospital, Tongji University School of Medicine, Shanghai, 200065 P.R. China

**Keywords:** Lung adenocarcinoma, Radiotherapy, Sensitivity, Fe_3_O_4_ magnetic nanoparticles, Baculoviral IAP repeat containing 5, Oligodeoxynucleotide antisense, Death receptor 5

## Abstract

**Background:**

Radiotherapy is the mainstay treatment for lung adenocarcinoma, yet remains highly susceptible to resistance. Fe_3_O_4_ magnetic nanoparticles (MNPs) possess the ability to induce biological therapeutic effects. Herein, the current study set out to explore the effects of Fe_3_O_4_ MNPs on radiosensitivity of lung adenocarcinoma cells.

**Methods:**

Fe_3_O_4_ MNPs loaded with both negatively-charged small interfering RNA against baculoviral IAP repeat containing 5 (siBIRC5) and oligodeoxynucleotide antisense (AS-ODN) to generate co-delivery NPs, followed by evaluation. Gel retardation assay was further performed to determine the binding ability of Fe_3_O_4_ MNPs to AS-ODN/siBIRC5. The radiosensitizing effect of NPs on lung adenocarcinoma cells was determined in the absence or the presence of NPs or radiotherapy. A549 and H460 tumor-bearing mice were established, where tumor tissues were subjected to immunohistochemistry.

**Results:**

NPs were successfully prepared and characterized. BIRC5 expression levels were augmented in tissues of lung cancer patients. Fe_3_O_4_ MNPs enhanced the uptake of siBIRC5 and AS-ODN by lung adenocarcinoma cells. The presence of NPs under magnetic field reduced the BIRC5 expression and elevated the DR5 expression in lung adenocarcinoma cells. Lung adenocarcinoma cells treated with NPs exhibited inhibited tumor cell migration and increased DNA damage. After magnetic field treatment, tumors were better suppressed in the tumor-bearing mice treated with NPs, followed by radiotherapy.

**Conclusion:**

Findings obtained in our study indicated that Fe_3_O_4_ MNPs-targeted delivery of siBIRC5 and AS-ODN enhances radiosensitivity, providing an innovative solution for the current clinically existing lung adenocarcinoma patients with radiotherapy resistance with a low risk of toxicity.

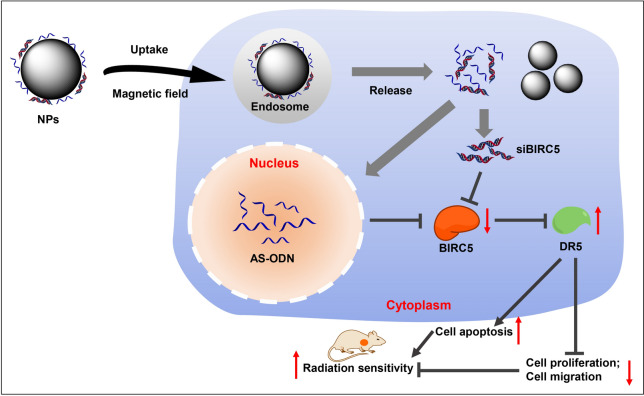

**Supplementary Information:**

The online version contains supplementary material available at 10.1186/s12967-021-02971-7.

## Background

Lung adenocarcinoma is the leading cause of cancer-related mortality, accounting for approximately 1.8 million deaths across the world in 2020, with high mortality rate reported [1–3]. There have been great advancements in the treatment of lung adenocarcinoma, amounting to chemotherapy, targeted therapy and radiotherapy in the current day [4]. Radiotherapy remains one of most common treatments for lung adenocarcinoma due to its ability to reduce metastasis occurrence, yet radiosensitivity encountered during the course of radiotherapy remains a limiting factor for achieving higher therapeutic effects [5]. Therefore, it would be prudent to explore molecular mechanisms underlying radiosensitivity of lung adenocarcinoma to enhance the efficacy of the treatment.

Nanomaterials (NPs) have been increasingly used in biomedical fields, especially in healthcare, which can be coupled with various antibody and luminescent labeling [6]. Due to their small size (100 nm) and surface paintability, NPs can also serve as effective tracers in cancer diagnosis with therapy [7]. In particular, 18 nm Fe_3_O_4_ magnetic nanoparticles (MNPs) are known to exhibit better magnetic particle and magnetic resonance imaging properties, which is designed for precision imaging and cancer therapy [8]. More interestingly, a recent study highlighted that MNPs can serve as a participant in a better efficacy against lung adenocarcinoma as a drug target [9]. Meanwhile, MNPs further serve as contributors to nanotriggers, which possess the ability to control cell function [10]. On the other hand, Baculoviral IAP repeat containing 5 (BIRC5) is up-regulated in lung adenocarcinoma cells and tissues, wherein radiosensitivity was found to influence the dependance on BIRC5 expression [11]. Moreover, genes can be inhibited by antisense oligodeoxynucleotide (AS-ODN) or by small interference RNA (siRNA), and previously been highlighted as a promising treatment avenue for lung adenocarcinoma [12]. Besides, death receptor 5 (DR5) is known to trigger cell death in various tumor cells [13], and further regarded as a prognostic biomarker for non-small cell lung adenocarcinoma [14]. However, the relationship among BIRC5, AS-ODN, and DR5 remains elusive and warrants further investigation. In the current study, we presumed that Fe_3_O_4_ MNPs may deliver BIRC5 and AS-ODN to tumor cells, which may be applied in radiotherapy, and set out to perform a series of experiments to investigate the molecular mechanism underlying small interfering RNA against BIRC5 (siBIRC5) in lung adenocarcinoma, hoping to discover some promising strategy to increase the radiosensitivity.

## Materials and methods

### Ethical statement

The current study was approved by the clinical research Ethics Committee (PR003/13) of Tongji University School of Medicine, and conformed with the guidelines of the *Declaration of Helsinki*. Signed informed consents were obtained from all participants prior to specimen collection. Animal experiments were performed in accordance with the standard of the Guide for *the Care and Use of Laboratory animals* published by the National Institutes of Health. Animal experiment protocols were reviewed and granted by the animal ethics committee of Tongji University School of Medicine. Extensive efforts were made to minimize the suffering of the included animals.

### ***Synthesis of Fe***_***3***_***O***_***4***_*** MNPs***

Fe_3_O_4_ MNPs were synthesized with the help of an oxidative hydrolysis method. First, to synthesize Fe_3_O_4_, FeCl_3_ deionized aqueous solution (1.66 g; 20 mL) was mixed with FeCl_2_ 4H_2_O (1.00 g; 20 mL) in deionized water. A black precipitate was observed after the addition of NH_4_OH solution (25%, 20 mL). The solution was stirred for 30-min to complete the reaction. Next, the solution was placed on a magnet, which allowed the magnetite to be absorbed to the surface. After the reaction solution was poured off, the magnetite dispersion was added with oleic acid solution in hexane (2.5–10 wt% solution in 20 mL hexane) under constant stirring. Concentration was subsequently performed by evaporation of hexane from the dispersion, and a black magnetite concentrate was obtained. Meanwhile, a stannous octoate catalyst was used for the polyethylene glycol lactide (PEG-LAC) copolymerization reactions. A total of 1.00 g lactide and 0.2 g PEG2000 were mixed in 9.00 mL toluene containing 0.005 g stannous octoate. The reaction balloon was then placed in a hot oil bath (140 °C) to react for 6 h. After the reaction mixture was cooled at room temperature, the copolymer was precipitated with icy diethyl ether liquid and dried in a vacuum oven. To cover the magnetite core surface with PEG-LAC layer, 20 mL of magnetite hexane dispersion was added to 20 mL of copolymer aqueous solution (1 wt% copolymer). Afterwards, the mixture was sonicated for 4 h to transfer the particles from the hexane top layer to the aqueous bottom layer. The residual dispersion in the aqueous phase was then dialyzed using a dialysis bag (1200) and lyophilized. To cover the chitosan layer on the surface of the previously synthesized particles, 0.05 g magnetite particles were dissolved in 1 mL dichloromethane, and then dripped onto an aqueous solution containing 0.2 mL acetic acid and 0.05 g chitosan. After overnight stirring, the solution was filtered through a 0.22 mm filter and lyophilized. Additionally, 0.02 g branched polyethyleneimine (PEI; 25 kDa) was dissolved in 10 mL deionized water, and added with the same amount (0.02 g) of product from the preparation of chitosan layer while maintaining stirring. Finally, the mixture was placed on a stirrer for 24 h.

### ***Characterization of Fe***_***3***_***O***_***4***_*** magnetic materials***

Fourier transform infrared reflection (FTIR) spectroscopy analysis was performed for Characterization of Fe3O4 magnetic materials. The samples (2 mg) and 200 mg potassium bromide were mixed and ground for 3 min. The mixture was then pressed into pellets for measurement. FTIR spectra were recorded using a FTIR spectrophotometer (Bruker tensor 27 spectrometer, Billerica, MA, USA) at the range of 400–4000 cm^−1^.

### Synthesis of NPs

The experimental materials for the synthesis of NPs were provided by Dharmacon (Lafayette, CO, USA). Scrambled control FAM-siBIRC5-NC (5′-CAGUCGCGUUUGCGACUGGUUdTdT-3′; 3′-dTdTGUCAGCGCAAACGCUGACCAA-5′) and FAM-siBIRC5 (5′-GGCUGGCUU CAUCCA-CUG-CdTdT-3′; 3′-dTdTCCGACCGAAGUAGGUGACG-5′) were synthesized by Integrated DNA Technologies (Coralville, IA, USA). In addition, FAM-AS-ODN (anti-sense: 5′-CCCAG-CCTTCCAGTCCCTTG-3′) and FAM-AS-ODN-NC (sense: 5′-CAAGGGACTGGAAGGCTGGG-3′) were purchased from Dharmacon (Lafayette, CO, USA).

To synthesize the Fe_3_O_4_ MNPs loaded with siBIRC5 solution and AS-ODN, the solution of siBIRC5 (100 ng) in Opti-minimum essential medium (MEM) and AS-ODN (200 ng) was added to the Fe_3_O_4_ MNPs. Subsequently, NPs were prepared after vortexing the resulting solution for 10 s and a 30-min incubation at room temperature.

### Size morphology characterization of NPS

NPs size and morphology were observed and analyzed with the help of transmission electron microscopy (TEM, Philips, Eindhoven, the Netherlands) and scanning electron microscopy (SEM, Philips, XL30 microscope instrument, the Netherlands). Particle size was analyzed using the ImageJ software. In addition, Zeta potential and hydrodynamic size of NPs were analyzed by dynamic light scattering (DLS, Mastersizer 2000, Malvern, Worcestershire, UK). NPs prepared with different mass ratios (Fe_3_O_4_ MNPs: siBIRC5: AS-ODN) were subjected to 3% agarose gel electrophoresis at 90 mV for 45 min, after which the gels were stained with ethidium bromide solution and observed under a GelDoc imaging system (UV doc-008, UVP, Upland, CA, USA).

### Clinical samples

Fresh squamous cell lung adenocarcinoma tissues and adjacent non-tumor lung tissues were collected from patients during resection surgery performed at the Tongji University School of Medicine. Histological typing was performed by the pathology department of the Tongji University School of Medicine. All human tissues were stored in RNAlater™ Medium, and stored in liquid nitrogen before processing. The selected samples were subsequently subjected to reverse transcription quantitative polymerase chain reaction (RT-qPCR). The expression patterns of BIRC5 were detected by Western blotting.

### Cell culture and transfection

Lung adenocarcinoma cells A549 (CCL-185, ATCC, Manassas, VA, USA) and H460 (HTB-177, ATCC, Manassas, VA, USA) were cultured in Dulbecco’s modified eagle medium (DMEM; CAT#01–055, Biological industries, Beit HaEmek, Israel). Meanwhile, human embryonic lung fibroblasts HFL-1 (CC-Y1584, EK-Bioscience, Shanghai, China) were cultured in Ham’s F-12 (CAT#01–095, Biological industries). All media were then supplemented with 10% fetal bovine serum (FBS; GIBCO™, CAT# 10270106, Life Technologies, San Jose, CA, USA), 100 units/mL penicillin, 100 µg/mL streptomycin, and 2 mM L-glutamine, which were all purchased from Biological industries (Kibbutz Beit Haemek, Israel). Non-essential amino acids (NEAA; CAT# X0557, 1: 100, Biowest, Logan, Utah, USA) were employed for HFL-1 culture. Afterwards, the cells were cultured in a humidified incubator (Thermo Fisher Scientific Inc., Waltham, MA, USA) at 37 °C with 5% CO_2_.

A549 cells were transfected with the HA-BIRC5 expression vector (pcDNA3-HA-BIRC5) and Myc-DR5 expression vector (DR5 Myc-tag) or corresponding empty vector (pcDNA) following the instructions of the Lipofectamine 2000 transfection reagent (CAT# 11668019, Invitrogen, Carlsbad, CA, USA). Cells were transfected with 1 μg of pcDNA3-HA-BIRC5 and DR5 Myc-tag or the corresponding empty vector pcDNA. After a 24-h period of transfection, the cells were treated with NPs for subsequent analyses.

### Western blotting

To detect the expression patterns of related proteins, highly efficient radio immunoprecipitation (RIPA) lysis buffer (R0010, Solarbio, Beijing, China) was adopted to extract the total protein content from the cells or tissues according to the manufacturer’s instructions. The supernatant was collected after 15-min lysis at 4 °C and 15-min centrifugation at 12,000 rpm. Protein concentration in the samples was subsequently determined using a bicinchoninic Acid (BCA) Kit (20201ES76, Yeasen Biology, Shanghai, China). Next, the proteins were mixed with sodium dodecyl sulfate (SDS) loading buffer after quantification, and samples were incubated at 10 °C for 5 min and cooled down naturally at room temperature. Samples and protein markers were then separated using SDS–polyacrylamide gel electrophoresis (PAGE; Solarbio). After electrophoresis, the gel was rinsed with deionized water and then transferred onto a polyvinylidene fluoride membrane. After the transfer, the membranes were blocked with 5% skimmed milk in tris-buffered saline tween (TBST) for 1 h at room temperature. To visualize the proteins on the membranes, the membranes were incubated with mouse anti-BIRC5 (dilution ratio of 1: 500, sc-17779, Santa Cruz biotechnology, CA, USA), mouse-anti-DR5 (dilution ratio of 1: 500, sc-166624, Santa Cruz biotechnology), and mouse anti-human caspase-3 antibodies (dilution ratio of 1: 500, sc-56053, Santa Cruz biotechnology) overnight at 4 °C. The following day, the membranes were then incubated with the horseradish peroxide (HRP) conjugated secondary antibody (dilution ratio of 1: 4000; Southern biotech, Birmingham, Alabama, US) for 1 h at room temperature. Afterwards, the membrane was detected using an enhanced chemiluminescence immunoblotting detection kit. In addition, mouse anti-β-actin antibody (dilution ratio of 1: 2000, sc-8432, Santa Cruz biotechnology) was treated as described previously. For absorbance analysis, scanned photographs were quantified using the AlphaEasy FC software (Alpha Innotech, San Leandro, CA, USA). Each experiment was repeated three times to obtain the mean value.

### RT-qPCR

Total RNA content was extracted the cells or tissues using RNeasy Mini kits (CAT#74104, Qiagen, Hilden, Germany) following the instructions to determine the transcription expression levels of genes. Complementary DNA (cDNA) was then reverse-transcribed with the help of a reverse transcription kits (Promega, Madison, WI). Subsequently, mRNA expression levels of related factors were determined using an ABI PRISM 7500 sequence detection system (Applied Biosystems, Foster City, CA). All primer sequences are shown in Additional file 3: Table S1.

### Cellular fluorescence imaging

To explore the uptake of siBIRC5 and AS-ODN, the cells were seeded in a 24-well culture plate and incubated for 12 h. A magnet (magnetic field: 0.5 T) was placed under the center position of the culture plate, and then incubated with AS-ODN (concentration of 200 nM) and siBIRC5 (concentration of 100 nM). Sense-ODN and scrambled siBIRC5 were transfected performed using the Lipofectamine 3000 transfection reagent (l300001, Invitrogen) as a positive control. FAM labeling was employed to observe the cellular uptake of siBIRC5 and AS-ODN with the help of fluorescence microscopy (observer A1, Carl Zeiss, Oberkochen, Germany). Fe_3_O_4_ MNPs loaded siBIRC5 as p-siBIRC5, while Fe_3_O_4_ MNPs loaded AS-ODN as p-AS-ODN. Simultaneously, sense-ODN and scrambled siBIRC5 were employed as the negative control (NC).

### Immunofluorescence staining

Five sterile round coverslips (1 cm × 1 cm) were placed on 6-well plates. Cells (1 × 10^5^ per well) were then seeded into each well. Following treatment with each preparation and magnetic field (0.5 T), the cells were fixed with 4% paraformaldehyde for 20 min, and then permeabilized with 0.2% Triton X-100 for 10 min. Next, the cells were blocked with 3% horse serum for 1 h at room temperature, and incubated with mouse anti-BIRC5 and mouse-anti-DR5 primary antibodies for 1 h. Afterwards, the cells were incubated with the anti-mouse secondary antibody labeled with tetramethylrhodamine for 1 h. Later, the cells were mounted with DAPI (Sigma Aldrich Co., St Louis, MO, USA) before imaging with a confocal microscope.

### γ-H2AX immunofluorescence staining

Cells were seeded in a 96-well plate with transparent black bottom (Corning, NY, USA) at 10^4^ cells per well, and incubated overnight at 37 °C in 5% CO_2_. Next, the cells were assigned into the control group, the NPs group (siBIRC5 with concentration of 500 nM, AS-ODN with concentration of 1000 nM), the RT group, and the NPs + RT group. After 24 h, the cells were fixed with 4% paraformaldehyde for 20 min. Fixed cells were then permeabilized with 0.1% Triton X-100 in PBS for 5 min, blocked with 1% bovine serum albumin (BSA) in PBS (blocking solution) for 30 min, and incubated overnight at 4 °C with mouse monoclonal anti-γ-H2AX antibody (dilution ratio of 1: 400 in blocking solution). Later, the cells were incubated in dark conditions with the FITC conjugated anti mouse antibody (dilution ratio of 1: 200 in blocking solution) for 1 h at room temperature and counterstained with 0.1 µg/mL DAPI for 1 min. Fluorescence pictures were taken on a Nikon A1R spectral confocal microscope and cytation 3 imaging multimode plate reader (BioTek, Winooski, VT, USA).

### Flow cytometry

Cellular uptake efficiency was determined. Following treatment with each preparation and magnetic field (0.5 T), the cells were rinsed twice with cold PBS. Next, the cells were trypsinized and then analyzed using a flow cytometer (BD facsverse, BD Biosciences, San Jose, CA, USA).

$${\text{Cellular}}\,{\text{uptake}}\,{\text{efficiency}}\,(\% )\, = \,\frac{{{\text{Cellular}}\,{\text{uptake}}\,{\text{of}}\,{\text{siBIRC5}}\,{\text{and}}\,{\text{AS - ODN}}}}{{{\text{siBIRC5}}\,{\text{and}}\,{\text{AS - ODN}}\,{\text{added}}\,{\text{to}}\,{\text{the}}\,{\text{cell}}}}$$

The expression patterns of DR5 were determined after cells were treated with each preparation and magnetic field (0.5 T) and trypsinized. Next, the cells were incubated with the PE conjugated anti-human-DR5 (CD262) antibody (Biolegend®, San Diego, CA, USA) for 30 min. The level of DR5 expression on the cell membrane was subsequently reflected by measuring the PE levels using flow cytometry (lsrfortessa™, BD Biosciences, San Jose, CA, USA).

Cell apoptosis was measured. Briefly, lung adenocarcinoma cell lines at the logarithmic phase of growth were seeded in 6-well plates at 2.0 × 10^5^ cells in each well. Four groups (control group, RT group, NPs group and the NPs + RT group) of cells were collected and counted. Annexin V-FITC/PI apoptosis detection kits were adopted for testing. Cell pellets were resuspended in 195 μL binding buffer and stained with 5 μL each of annexin V-FITC and PI staining solution for 10 min at room temperature in dark conditions. Flow cytometry was then performed on a FACScan system with CellQuest software. The apoptosis rate was calculated as follows: (number of apoptosis in each group / total number of cells in each group) × 100%. Three parallels were set for each group to obtain the mean value.

### Enzyme-linked immunoassay (ELISA)

To measure BIRC5 protein, we seeded cells (1 × 10^5^ per well) into 6-well plates. After cells were treated with each preparation and magnetic field (0.5 T), cells were treated with 200 µL of 0.5% Triton X-100 lysis buffer for 30 min in an ice bath. Cell lysates were collected, vortexed briefly, and then incubated on ice for an additional 15 min. Cell debris was removed by centrifugation at 2000 g for 5 min, diluted with 1% BSA in PBS and protein concentration was determined by Coomassie blue assay (Bradford method). A 10 mg sample of total protein was added to a 96-well plate precoated with capture antibody, and BIRC5 protein was determined by ELISA Kit (R&D systems, Minneapolis, MN, USA).

### 3-(4,5-Dimethylthiazol-2-yl)-2,5-diphenyltetrazolium bromide (MTT) assay

To evaluate cell proliferation, cells were seeded in 96-well plates at 5000 cells/well. The medium was removed the following day. Subsequently, NPs containing 0, 250, 500, 750, 1000 μM of siBIRC5 were incubated with cells under a magnetic field (0.5 T). After 24 h, the cells were irradiated at doses of 0 Gy (no radiation), 2 Gy, 4 Gy, 6 Gy, and 8 Gy, and incubation was continued for another 48 h. The wells were added with a total of 0.5 mg/mL MTT reagent and treated for 1 h, and then with dimethyl sulfoxide (DMSO, Serva electrophoresis GmbH, Heidelberg, Germany). The absorbance of the samples was measured at 570 nm using a synergy HTX microplate reader (BioTek).

### Plate clonogenic assay

To evaluate cell proliferation, cells with or without NPs (siBIRC5 with concentration of 500 nM; AS-ODN with concentration of 1000 nM) under a magnetic field (0.5 T), which were irradiated at room temperature with γ-ray at doses of 0, 2, 4, 6, and 8 Gy. Subsequently, using a ^137^Cs light source (Mark 1–68 irradiator, JL Shepherd & Associated, San Fernando, CA, USA), the cells were irradiated at a dose rate of 3.66 Gy/min and further incubated for another 6 h. Next, the cells were trypsinized and counted, and then the cells were cultured in drug-free medium to analyze the colony forming ability. After 2 weeks of culture, the cells were fixed and stained with PBS containing 4% formaldehyde and 0.05% crystal violet, and colonies with greater than 50 cells were counted. Three parallels were set for each group.

### Scratch test

To measure cell migration, cells were added in triplicate in 6-well plates and incubated until cell grew adherent to the wall. Cells were scratched using a 10 μL pipette. Subsequently, the control, the NPs (siBIRC5 with concentration 500 nM; AS-ODN with concentration 1000 nM), the radiotherapy (with radiation at 2 Gy dose), as well as the NPs + RT group were set up. Cells were first incubated in the presence or absence of NPs under a magnetic field (0.5 T), and then treated with or without radiation. After a 24-h period of incubation, the samples were observed using a phase contrast microscope.

### Xenograft tumor in nude mice

BALB/C female nude mice (aged 5–6 weeks old) were housed in a specific-pathogen-free grade animal room with room temperature of 25 °C and humidity of 70%, 12-h light/dark cycle with ad libitum access to water and food. Animal experiments were performed by subcutaneous injection of A549 and H460 cell suspensions (2 × 10^6^ cells/100 μL) into the right rear flank of mice, respectively. The experiment was conducted when the tumor volume grew to 100 mm^3^. Tumor volume = 0.5 × A^2^ × B (A = width, B = length).

### In vivo* radiotherapy sensitization*

To explore the effects of radiotherapy in vivo, A549 and H460 subcutaneous tumor bearing mice were randomly divided into the following four groups: (a) the control group (P-NC), (b) the NPs injection group, (c) the radiotherapy alone group (RT) and (d) the NPs combined with RT group (NPS + RT). Following intravenous injection of NPs (25 mg/kg/day), a magnet (magnetic field: 0.5 T) was fixed at the tumor site of the mice. After 24 h, radiotherapy (2 Gy/day) was performed for 5 consecutive days. Tumor volume (mm^3^) was measured every other day, and mouse body weight was recorded. Simultaneously, mice in the control group and the RT group were injected with 0.9% NaCl solution alone at the same time point. On the 30th day, the mice were euthanized, and the tumor tissues were removed for photographing and weight measurement.

Another group of mice were euthanized, and the tumor tissues from tumor bearing mice were removed on day 15 from the (a) control (P-NC), (b) NPs injection group, (c) radiotherapy alone group (RT) and (d) NPs combined with RT group (NPS + RT) for RT-qPCR, Western blotting, and immunohistochemical staining.

### Immunohistochemistry and Terminal deoxynucleotidyl transferase dUTP Nick-End Labeling (TUNEL) staining

For immunohistochemical staining, after deproteinization, hydration, and antigen retrieval, tumor tissue sections were treated with endogenous peroxidase in 0.3% H_2_O_2_ for 10 min. Next, the sections were blocked with PBS containing 1.5% blocking serum, and then incubated with the primary antibodies rabbit polyclonal anti-DR5, rabbit polyclonal anti-BIRC5, or rabbit monoclonal anti-Ki67 overnight at 4 °C. Later, the sections were added with appropriate amounts of goat anti-rabbit or anti-mouse secondary antibody working solution (ZSGB-Bio, Guangzhou, China) for 1 h incubation at 37 °C. Color was developed using diaminobenzidine (DAB) (ZSGB-Bio) for 3–5 min. Afterwards, the sections were counterstained with hematoxylin, while sections without incubation with primary antibody were regarded as the NC. The results were observed microscopically and evaluated by quantifying the staining intensity and the percentage of stained tumor cells. Expressions were analyzed by two independent investigators blinded to clinical data using a multi-headed microscope.

TUNEL staining was performed according to the manufacturer’s instructions (Roche Applied Sciences, Germany) and previously established methods [15].

### Evaluation of NP biocompatibility

To evaluate the NP biocompatibility, healthy BALB/c mice were injected with NPs (25 mg/kg/day), and changes in body weight were observed over 15 days. Another batch of healthy mice was taken, and blood samples were collected for liver and kidney function indexes and blood routine analysis on days 1, 7, and 15 after injection. Mice were euthanized on day 15. Major organs, including heart, lung, kidney, liver, and spleen, were excised, while tissues were fixed using formalin, embedded with paraffin, and then sectioned. Later, the Sections (4 μm) were stained with hematoxylin–eosin for histological examination.

### Statistical analysis

Statistical analyses were performed using the SPSS21.0 software (IBM SPSS statistics, Armonk, NY, USA). Measurement data were expressed as mean ± standard deviation, and two group data were compared following an unpaired design with normal distribution and homogeneous variance, using unpaired *t*-test. Data comparisons among multiple groups were performed by one-way analysis of variance (ANOVA) followed by Tukey’s post-hoc test. Survival was calculated using the Kaplan–Meier method. A value of *p* < 0.05 was regarded statistically significant.

## Results

### Successful preparation and characterization of NPS

Firstly, we verified the surface structure modification of prepared Fe_3_O_4_ MNPs by means of FTIR spectroscopy (Additional file 1: Fig. S1). The preparation steps and structural schematic of the whole MNPs are shown in Additional file 1: Fig. S1A. The characteristic functional groups of (B) Fe_3_O_4_ MNPs, (C) PEG-LAC, (D) Fe_3_O_4_-PEG-LAC chitosan, and (E) Fe_3_O_4_-PEG-LAC-chitosan-PEI confirmed the successful modification of each component of the MNPs. As shown Additional file 1: Fig. S1B_1_, the Fe_3_O_4_ MNPs spectrum exhibited a characteristic absorption band at 570 cm^−1^, which was an Fe–O bond, indicating that Fe_3_O_4_ MNPs were successfully synthesized. Additional file 1: Fig. S1B_2_ illustrates the infrared spectra for FTIR PEG-LAC. Absorption bands at 900–1200 cm^−1^ referred to C–O stretching vibrations, where bands between 1200 and 1500 cm^−1^ indicated the formation of ester bonds. Meanwhile, the strong band at 175 cm^−1^ evidenced the presence of carbonyl groups (C = O), while the bands at 3000 cm^−1^ and 3500 cm^−1^ were indicative of C–H and O–H stretching vibrations, respectively. The FTIR spectrum of Fe_3_O_4-_PEG-LAC-chitosan was shown in Additional file 1: Fig. S1B_3_, wherein the Fe_3_O_4_ characteristic band reappeared at 569 cm^−1^. The band shown here was the same as in Additional file 1: Fig. S1B except for the presence of two N–H stretching absorptions (NH_2_) at 2860 cm^−1^ and 2925 cm^−1^. As shown in Additional file 1: Fig. S1B_4_, though Fe_3_O_4-_PEG-LAC-chitosan-PEI presented with the same band, the intensity of the NH_2_ band was increased, while the intensity of other bands was decreased. We presumed this variation was attributed to the use of PEI for surface coating, which provided a higher positive charge through its amido resulting in a change in the spectrum.

After successfully preparing Fe_3_O_4_ MNPs, we adopted high positive charge distribution on the surface of Fe_3_O_4_ MNPs to load AS-ODN and siBIRC5 to obtain NPs. To further verify that AS-ODN and siBIRC5 could form stable complexes with Fe_3_O_4_ MNPs, we performed agarose gel electrophoresis after incubating 1 µg of siBIRC5 and 2 µg of AS-ODN with 1, 5, 10, and 20 µg of Fe_3_O_4_ MNPs. As shown in Additional file 1: Fig. S1C, stable nanocomplexes were formed when the mass ratio reached Fe_3_O_4_ MNPs: siBIRC5: AS-ODN = 5: 1: 2 and above. In addition, the unbound siBIRC5 bands disappeared from the surface of the gel. Thereafter, we used NPs prepared at a mass ratio of 5: 1: 2 (Fe_3_O_4_ MNPs: siBIRC5: AS-ODN) for subsequent experiments. We subsequently tested the particle size and zeta potential of the NPs. As shown in Additional file 1: Fig. S1D, the synthesized NPs exhibited dense spherical morphology, with an average diameter of 50 nm as illustrated by TEM imaging (Additional file 1: Fig. S1E). Moreover, SEM imaging (Additional file 1: Fig. S1F) confirmed that the morphology of the NPs was uniform and well-dispersed. Meanwhile, DLS showed that the particles with an average diameter of 46.28 nm and a size ≤ 100 nm were more advantageous and more readily taken up by cells for clinical applications. Furthermore, the results of electrical potential measurement verified that the surface of Fe_3_O_4_ MNPs was positively charged (+ 75 mV), which provided the preconditions for the formation of electrostatic nanomaterial NPs loaded with AS-ODN and siBIRC5, whereas the prepared NPs also showed a lower positive charge (+ 3.39 mV), which was favorable for cellular uptake.

### NPs improved the uptake of siBIRC5 and AS-ODN by tumor cells

**Fig. 1:**
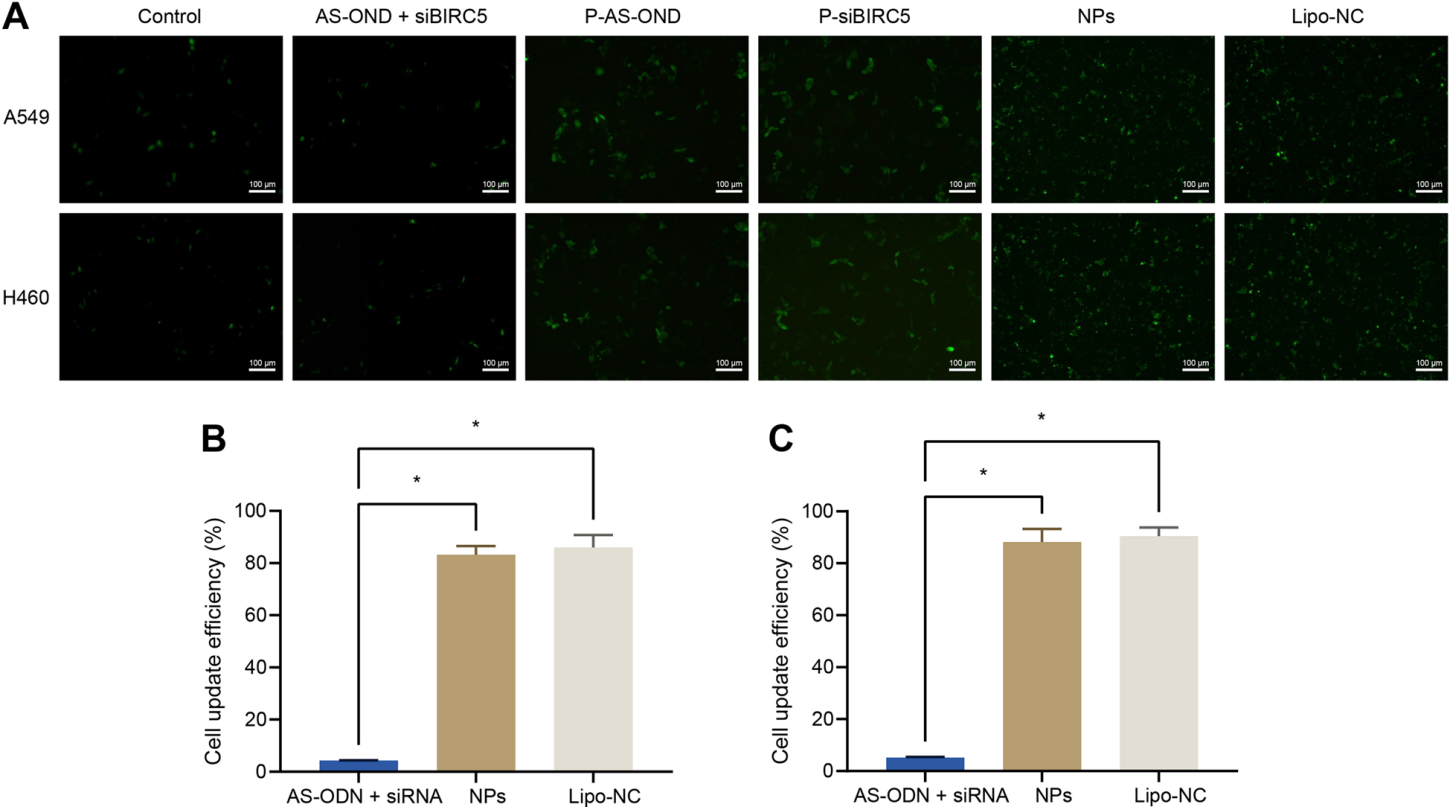
Fe_3_O_4_ MNPs carrier affect the uptake of siBIRC5 and AS-ODN by cells. **A** Delivery of siBIRC5 and AS-ODN into A549 and H460 cells by visualization of FAM-labeled siBIRC5 and AS-ODN by Fe_3_O_4_ MNPs under magnetic field. Lipofectamine 3000 (lipid) was used as a positive control for siBIRC5 and AS-ODN transfection (scale bars = 100 µm). **B** Fluorescence intensity of A549 cell lysates analyzed by fluorescence spectroscopy (λ_ex_: 494 nm; λ_em_: 519 nm). **C** Fluorescence intensity of H460 cell lysates analyzed by fluorescence spectroscopy (λ_ex_: 494 nm; λ_em_: 519 nm). * indicates *p* < 0.05

To verify whether Fe_3_O_4_ MNPs could enhance the uptake of siBIRC5 and AS-ODN by A549 and H460 cells, we incubated cells under a magnetic field with control PBS, free siBIRC5 mixed with free AS-ODN, Fe_3_O_4_ loaded with AS-ODN (P-AS-ODN), Fe_3_O_4_ loaded with siBIRC5 (P-siBIRC5), positive control Lipo-NC, and NPs, followed by fluorescence imaging. As shown in Fig. 1A, except for the PBS group and free siBIRC5 mixed with free AS-ODN group, obvious FAM green fluorescence was apparent in the cells of other groups, indicating that Fe_3_O_4_ MNPs effectively delivered siBIRC5 and AS-ODN to A549 and H460 cells. Meanwhile, the results of flow cytometry demonstrated that the mean fluorescence intensity of NPs treated A540 and H460 cells were about 30 times higher than those of free AS-ODN and siBIRC5 by delivery of Fe_3_O_4_ MNPs with AS-ODN and siBIRC5, while the uptake efficiency could reach 80% in both cells (Fig. 1B, C). Together, these findings indicated that the Fe_3_O_4_ MNPs carrier significantly improved the delivery efficiency of siBIRC5 and AS-ODN to A549 cells.

### BIRC5 is highly-expressed in lung adenocarcinoma patients and NPs inhibited BIRC5 expression in tumor cells

**Fig. 2:**
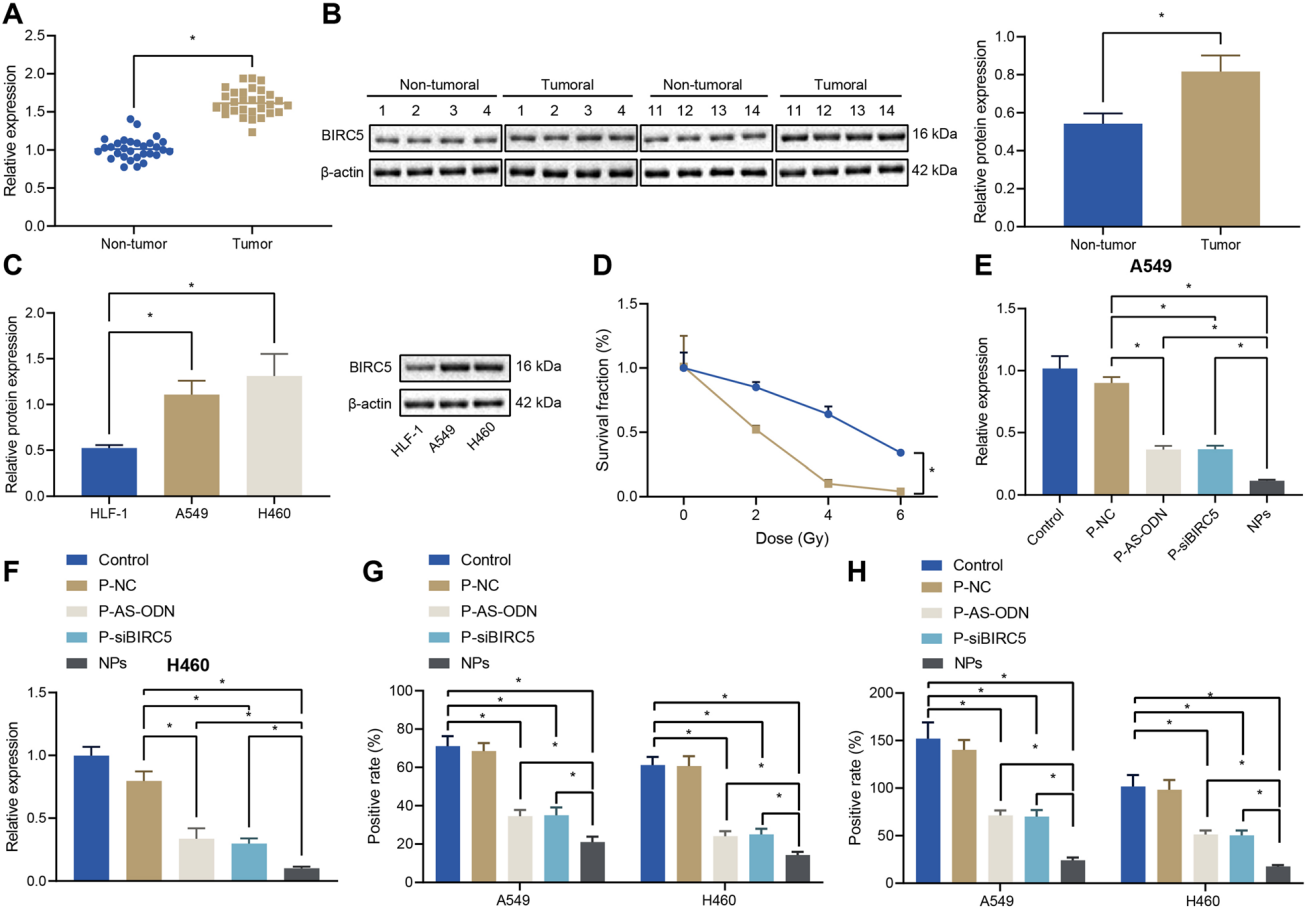
NPs decreases BIRC5 expression in tumor cells. **A** BIRC5 expression in tissues from lung adenocarcinoma patients and non-tumor tissues detected by RT-qPCR. **B** BIRC5 expression in tissues from lung adenocarcinoma patients and non-tumor tissues detected by Western blotting. **C** BIRC5 protein expression in different groups analyzed by Western blotting. **D** Colony formation of A549 and H460 cells. **E** BIRC5 mRNA expression quantified in A549 cells by RT-qPCR after different treatment. **F** BIRC5 mRNA expression quantified in H460 cells by RT-qPCR after different treatment. **G** Immunofluorescence staining after different treatment. BIRC5 positive-cells are counted. H, BIRC5 content quantified in A549 and H460 cells by ELISA after different treatment. * indicates *p* < 0.05

To further evaluate BIRC5 as a potential target for tumor therapy, we analyzed the expression patterns of BIRC5, and found that BIRC5 was up-regulated more than 19-fold in tumor tissues relative to non-tumor samples (Fig. 2A), and BIRC5 protein expression levels were much higher in the tumor samples than those in non-tumor samples (Fig. 2B). In addition, BIRC5 over-expression was observed both in A549 and H460 cells compared to non-tumor human lung fibroblast HFL-1 (Fig. 2C). Moreover, the results of clonogenic assay showed that the radiotherapy survival fraction of lung adenocarcinoma cells A549 was 0.84, and that of H460 cells was 0.44, indicating that A549 cells exhibited some radiation resistance relative to H460 cells (Fig. 2D).

**Fig. 3:**
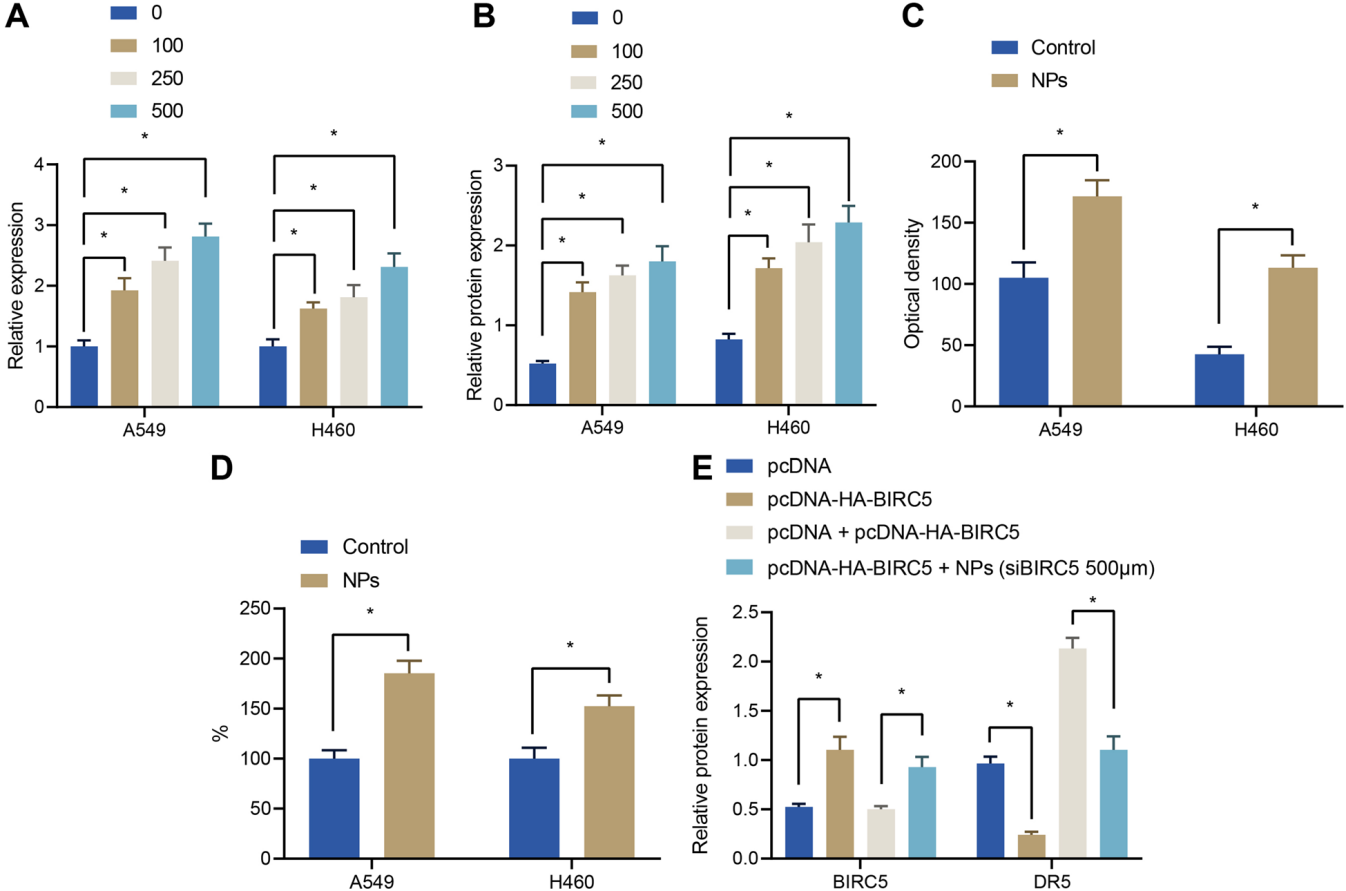
NPs increases DR5 expression by inhibiting BIRC5 level. **A** RT-qPCR validation of BIRC5 expression in lung adenocarcinoma cells treated with different concentrations of NPs (concentrations indicated as concentrations of siBIRC5). **B** Western blotting result of DR5 expression in lung adenocarcinoma cells treated with different concentrations of NPs. **C** DR5 expression detected by immunofluorescence staining after NPs treatment of lung adenocarcinoma cells for 24 h. **D** Lung adenocarcinoma cells labeled with PE-coupled anti-human DR5 (CD262) and the expression of DR5 in lung adenocarcinoma cells determined by flow cytometry. **E** Western blotting results of DR5 expression in A549 cells

Both siBIRC5 and AS-ODN could effectively inhibit BIRC5 expression. To verify the whether BIRC5 expression could be co-regulated by the co-delivery of siBIRC5 and AS-ODN by Fe_3_O_4_ MNPs, we incubated NPs with A549 and H460 cells under a magnetic field. As illustrated in Fig. 3E, F, since AS-ODN and siBIRC5 were equipped with a negative charge, it was difficult to permeate the cell membrane with poor cellular bioavailability. Moreover, AS-ODN and siBIRC5 exhibited insignificant down-regulation of BIRC5 mRNA expressions in A549 and H460 cells. Meanwhile, Fe_3_O_4_ MNPs (P-NC) containing sense-ODN and scrambled siBIRC5 exhibited only marginal effects. On the other hand, delivery of AS-ODN (P-AS-ODN) and siBIRC5 (P-siBIRC5) groups alone using Fe_3_O_4_ MNPs down-regulated BIRC5 mRNA expression levels by 37% and 30% in A549 cells and by 35% and 31% in H460 cells, respectively. However, the use of NPs simultaneously loaded with AS-ODN and siBIRC5 down-regulated BIRC5 mRNA levels by 60% and 58% in A549 and H460 tumor cells, respectively. As shown in Fig. 2G, simultaneous delivery of AS-ODN and siBIRC5 by Fe_3_O_4_ MNPs could also down-regulate BIRC5 protein expression levels in A549 and H460 cells, showing only 36% and 38% BIRC5 positive cells, respectively. However, there were 51% and 58% BIRC5 positive cells in A549 cells and 52% and 60% BIRC5 positive cells in H460 cells treated with P-AS-ODN and P-siBIRC5, respectively. Furthermore, the results of ELISA were consistent with the results of immunofluorescence staining. As shown in Fig. 2H, the content of BIRC5 in the cells was found to be the lowest in the NPs group, and the above results indicated that Fe_3_O_4_ MNPs effectively and simultaneously delivered AS-ODN and siBIRC5 to lung adenocarcinoma tumor cells, to reduce the BIRC5 expression in tumor cells via co-inhibition.

### NPs promoted DR5 expression by suppressing BIRC5 expression

Furthermore, we incubated NPs at different concentrations with A549 and H460 cells under a magnetic field, and found that DR5 expression levels in tumor cells were also increased with increasing concentrations of NPs (Fig. 3A, B). As expected, the results of immunofluorescence staining and flow cytometry revealed that A549 and H460 cells treated with NPs presented with increased DR5 fluorescence signal and increased DR5 expression levels (Fig. 3C, D). To further verify that up-regulation of DR5 was mediated through NPs-inhibited BIRC5 expression, we transfected A549 cells with BIRC5 cDNA expression vector, the results of which demonstrated that BIRC5 blocked the up-regulation of DR5 by NPs (Fig. 3E), indicating that NPs inhibited BIRC5, and thereby up-regulated the expression of DR5.

### *NPs play promoting role in sensitivity to radiotherapy *in vitro

**Fig. 4:**
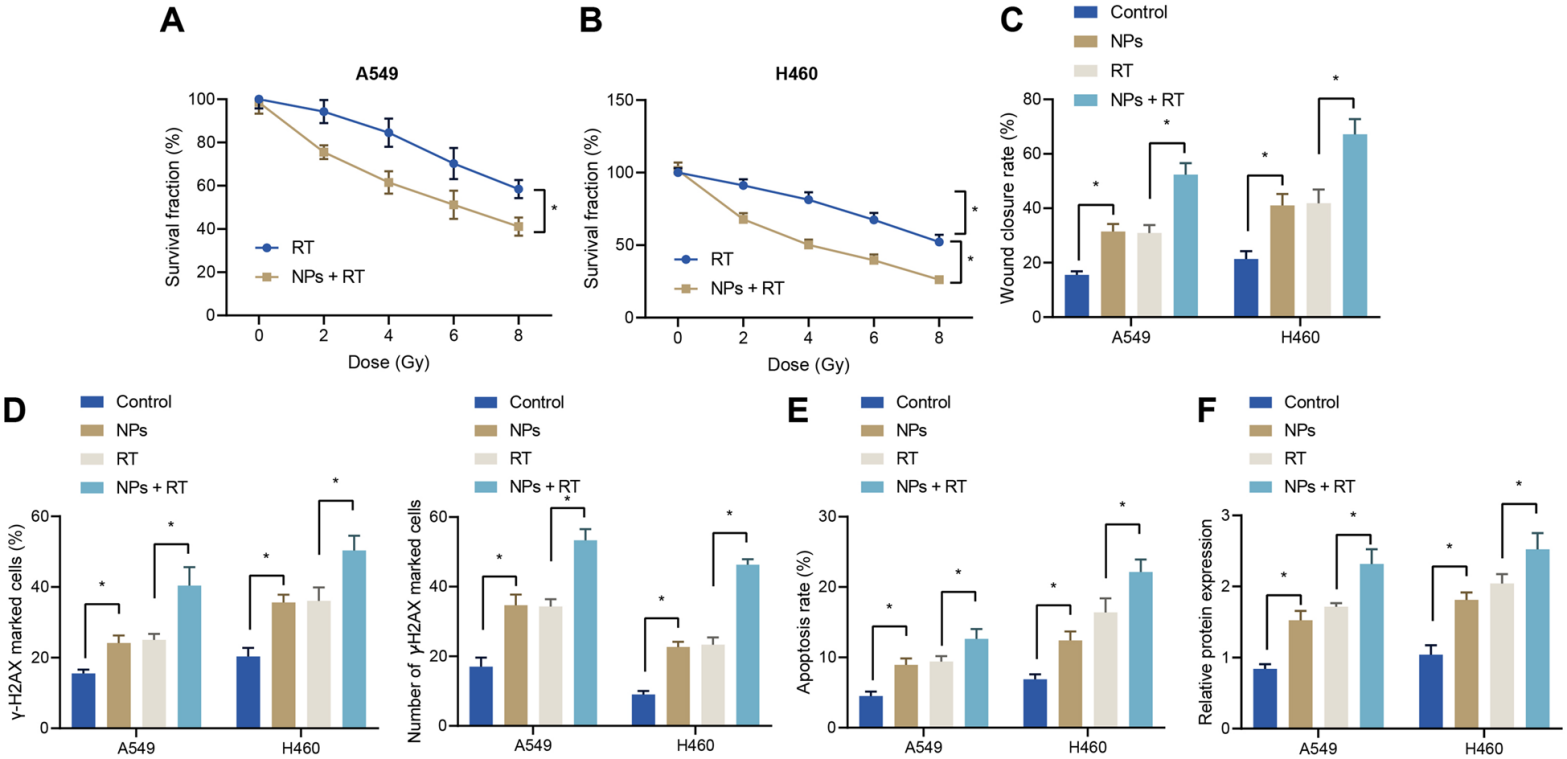
NPs facilitate sensitivity of lung adenocarcinoma cells to radiotherapy. **A** Colony survival analysis of A549 cells treated with or without NPs. **B** Colony survival analysis of H460 cells treated with or without NPs. **C** Cell migration studied by scratch assay. **D** γ-H2AX immunofluorescence staining of lung adenocarcinoma A549 and H460 cells in γ-H2AX-positive cells percentage and number of γ-H2AX foci in γ-H2AX-positive cells. **E** Apoptosis rate of lung adenocarcinoma cells in different treatment groups analyzed by flow cytometry. **F** Caspase-3 expression in lung adenocarcinoma cells of different treatment groups determined with Western blotting. * indicates *p* < 0.05

Additionally, we investigated the radiosensitization of NPs in vitro. As shown in Additional file 2: Fig. 2, both A549 and H460 cell viability exhibited a gradually decreasing trend with increasing NPs concentration and radiation dose, while the killing effect of NPs combined with radiotherapy on tumor cells was significantly promoted compared with radiotherapy alone group and NPs group. As shown in Fig. 4A, B, the surviving fraction (SF2) of A549 and H460 cells at different Gy significantly decreased following treatment with NPs, indicating that NPs enhanced the sensitivity of lung adenocarcinoma cells to radiotherapy. Moreover, compared with the radiotherapy alone group, the lung adenocarcinoma cell migration rate was found to be obviously decreased in the NPs + RP group, demonstrating that NPs further inhibited the migration effect of radiotherapy on tumor cells (Fig. 4C). Meanwhile, lung adenocarcinoma cells in the NPs + RT group showed an increase in the percentage of γ-H2AX positive cells. In addition, the number of γ-H2AX lesions was also increased in the NPs group compared to radiotherapy alone group, indicating that NPs significantly increased DNA damage to tumor cells by radiotherapy (Fig. 4D). Furthermore, lung adenocarcinoma cell apoptosis was observed to be increased in the NPs group, the RT group, and the NPs combined with radiotherapy (NPS + RT) group, with the most apparent apoptosis effect in the NPs + RT group (Fig. 4E). As shown in Fig. 4F, compared with the control group, the expression levels of Caspase-3 in the NPs group and the RT group were higher than those in the control group, while the expression levels of Caspase-3 were most elevated in the NPs + RT group. Altogether, these findings indicated that NPs improved the sensitivity of tumor cells to radiotherapy.

### *NPs promoted sensitivity to radiotherapy through BIRC5/DR5 *in vivo

**Fig. 5:**
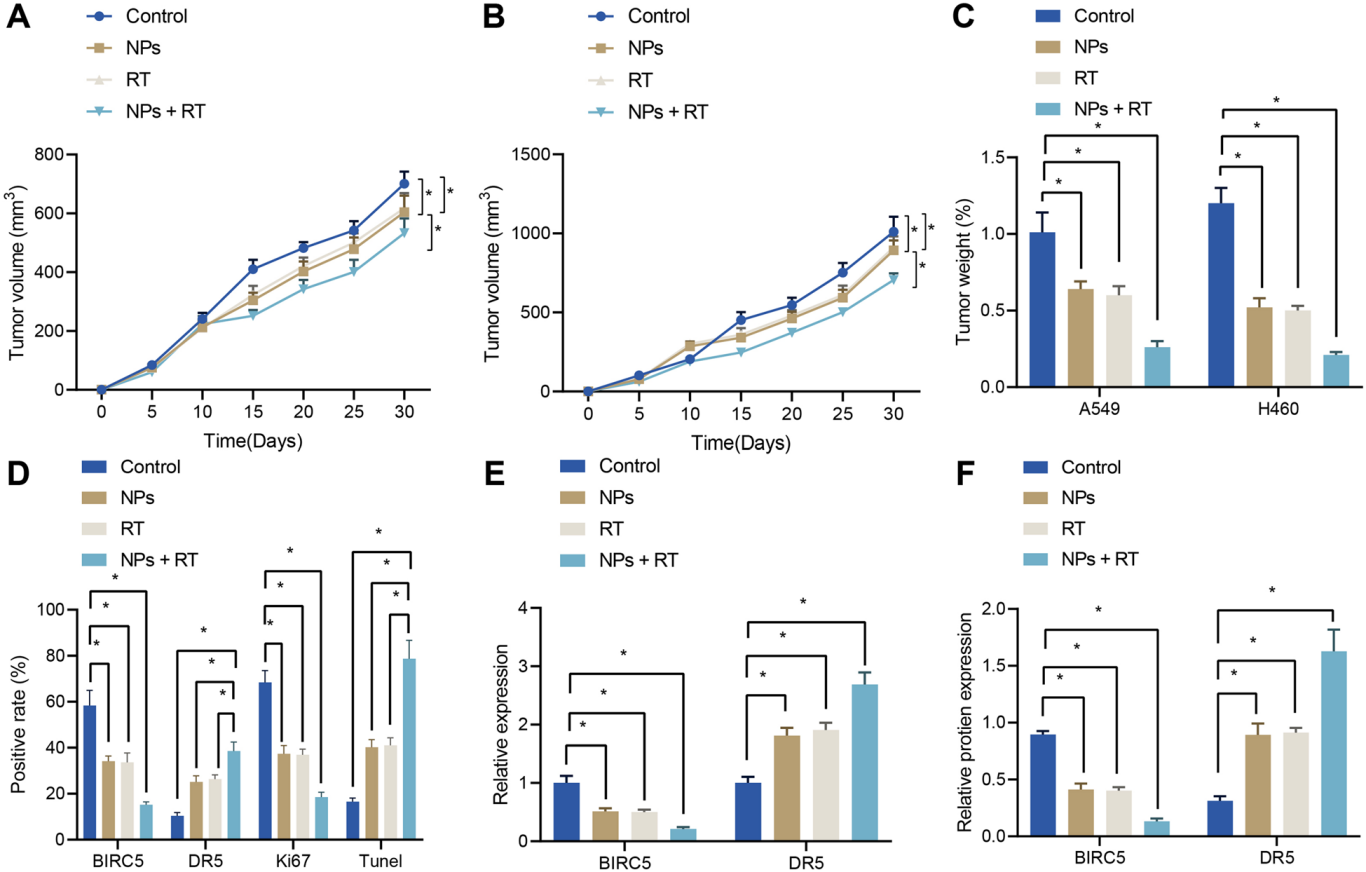
NPs promote sensitivity to radiotherapy in vivo*.* **A** Tumor mass of mice bearing tumors. **B** Tumor size of mice bearing tumors. **C** Tumor tissue weight of mice on day 30. **D** Immunohistochemical staining and TUNEL staining of BIRC5, DR5, and Ki67 in different treatment groups. **E** RT-qPCR results of the BIRC5 and DR5 expression in tumor tissues of mice injected with A549 cells. **F** Western blotting results of the BIRC5 and DR5 expression in tumor tissues of mice injected with A549 cells. *p < 0.05 compared with the control group; #*p* < 0.05 compared with the RT group

After verifying that the NPs conferred radiosensitizing effects in vitro, we established subcutaneous A549 and H460 tumor xenografts in mice to directly verify whether the NPs loaded with siBIRC5 and AS-ODN could also exhibit radiosensitizing effects in vivo. After the mice were intravenously injected with NPs and maintained under a magnetic field, the tumor bearing mice were subjected to radiotherapy with tumor growth of mice recorded. In addition, the tumor tissues were weighed. It was found that the NPs combined with RT group presented with reduced weight and stronger growth inhibition effects on the subcutaneous tumors of A549 and H460 cells (Fig. 5A–C). Overall, these results suggested that the NPs possessed a certain radiosensitizing effect on lung adenocarcinoma subcutaneous tumors.

To further demonstrate the radiosensitizing effects of NPs in vivo by inhibiting BIRC5 to up-regulate DR5, we subjected tissues of mice bearing subcutaneous tumors to radiotherapy alone and NPs combined with radiotherapy. It was observed that compared with the radiotherapy alone group, the BIRC5 positive cells were decreased, whereas the DR5 positive cells increased significantly in the NPs combined with radiotherapy group. Additionally, the number of Ki67 positive cells in the NPs combined with radiotherapy group was found to be significantly lower than that in the RT group, accompanied by increased number of TUNEL positive cells, indicating that NPs enhanced tumor apoptosis and inhibited tumor proliferation after radiotherapy (Fig. 5D). The results of RT-qPCR and Western blotting further validated that BIRC5 expression levels were obviously down-regulated, while the expression levels of DR5 were distinctly up-regulated in the NPs + RT group (Fig. 5E–F).

### NPs demonstrate great biological compatibility

**Fig. 6:**
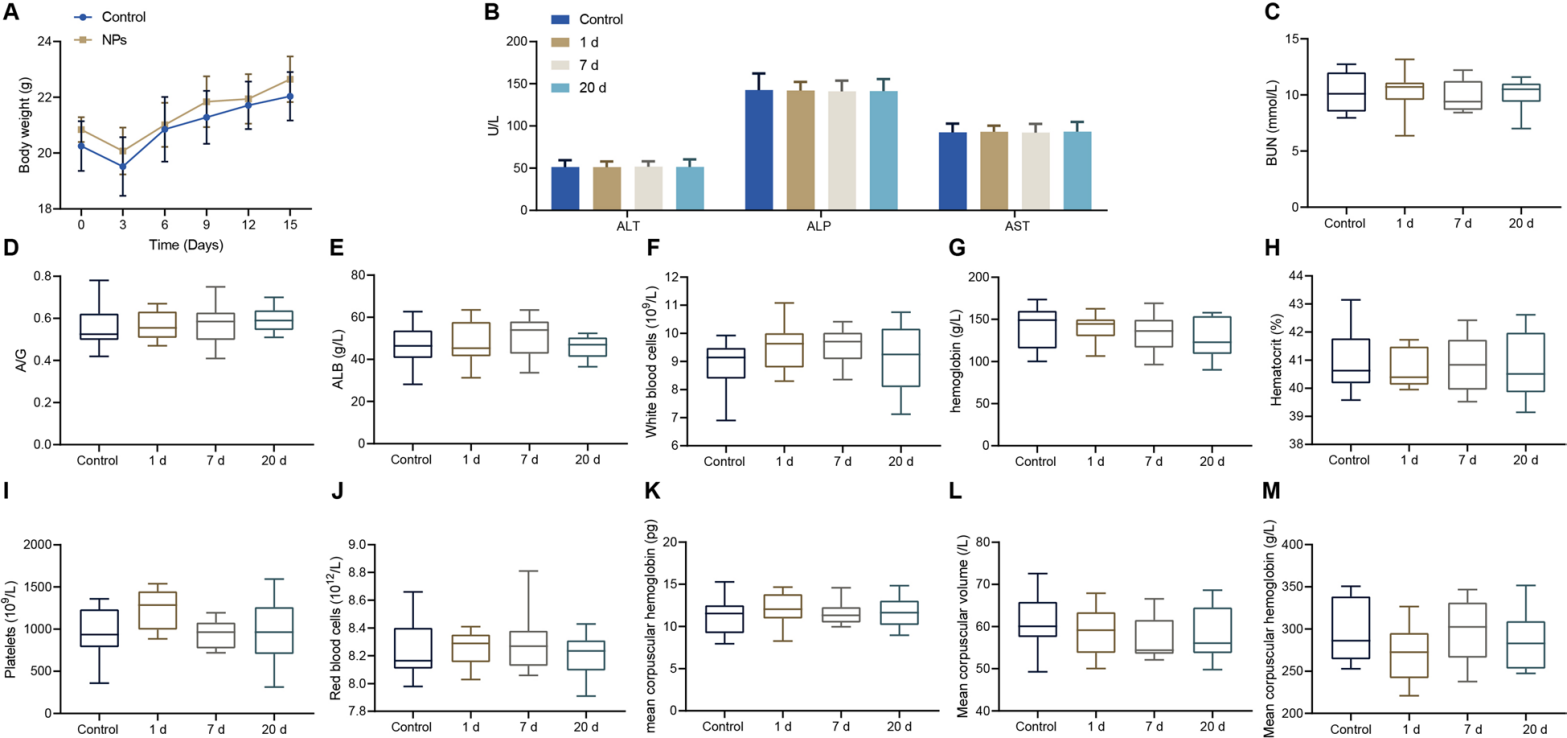
NPs have no obvious side effects on the murine model. **A** Body weight changes in mice after intravenous injection with or without NPs. **B–M** Blood biochemistry (**B–E**) and routine blood analysis (**F**–**M**) in mice treated with or without NPs at day 1, 7, and 15 after intravenous injection

Potential toxicity in vivo remains a significant concern for the widespread application of nanomaterials in biomedicine. Therefore, we systematically investigated the potential toxicity in vivo of NPs by monitoring the body weight, blood indices, and histological examination of mice. Following intravenous injection of NPs to healthy mice, the body weight was monitored in real time. As shown in Fig. 6A, both mice injected with NPs and the control showed a slight rise in body weight, indicating that NPs did not cause obvious systematic toxicity to mice. In addition, after intravenous injection of NPs to mice, we collected mice blood samples for analysis at day 1, 7, and 15 after injection. As reflected by Fig. 6B-E, serum biochemical parameters including liver function markers [alanine aminotransferase (ALT), alkaline phosphatase (ALP) and aspartate aminotransferase (AST)] and renal function markers urea nitrogen (BUN) and albumin/globulin ratio were all found to be within normal ranges. We further investigated routine blood examination, including white blood cells, red blood cells, hemoglobin, mean corpuscular volume, mean corpuscular hemoglobin, mean corpuscular hemoglobin concentration, platelet count, and mean corpuscular hemoglobin. Compared with the control group, all the aforementioned parameters were also observed to be within the normal range (Fig. 6F–M). Altogether, these findings indicated that NPs exerted no obvious side effects on mice.

## Discussion

Radiation therapy remains a mainstay for treating malignant tumors with approximately 70% of cancer patients requiring radiation therapy, yet resistance to radiation still requires solutions [16]. Currently, patients with lung adenocarcinoma are primarily treated with surgical means, radiotherapy and chemotherapy, but still face the problem of chemotherapy resistance resulting in poor prognoses in patients. In order to tackle radiotherapy sensitivity of lung adenocarcinoma, we prepared Fe3O4 MNPs loaded with siBIRC5 and AS-ODN. By characterizing, we successfully constructed Fe3O4 MNPs/siBIRC5/DR5, and verified to enhance radiosensitivity in vivo and in vitro. The mechanism of radiosensitization effect of Fe3O4 MNPs was primarily achieved by inhibiting BIRC5 and thereby up-regulating DR5 to enhance in vivo radiosensitivity, with lower risk of toxicity.

Following the preparation of Fe_3_O_4_ MNPs, we further verified the surface structure modification. The average diameter of Fe_3_O_4_@Au-C225 composite-targeted MNPs is known to be around 46 nm, which resides in the narrow size distribution as previously illustrated by laser particle size analysis [17]. Similarly, findings obtained in our study revealed that the average diameter of Fe_3_O_4_ MNPs-siBIRC5-AS-ODN was 46.28 nm, which is in accordance with the previous study and guarantees the feasibility of our study. Meanwhile, adsorption is regarded as an electrostatic interaction between the negative charge and positive charge group, which forms a solid combination with the help of a stable chemical bond [18]. These evidences highlight the ability of cells to uptake the prepared NPs. Additional experimental findings in our study displayed that NPs promoted the uptake of siBIRC5 and AS-ODN by lung adenocarcinoma cells, which suggests that Fe_3_O_4_ MNPs could effectively deliver siBIRC5 and AS-ODN into A549 and H460 cells. Further in line with our findings, a prior study illustrated that the anticancer nanocomposite (Fe_3_O_4_-PEG-GA) can also exert certain effects on human lung adenocarcinoma cells A549 [19].

Subsequent experimentation in our study revealed that NPs exerted a suppressive effect on BIRC5 expression in lung adenocarcinoma cells. As a member of the inhibitor of apoptosis protein family, BIRC5 is known to be up-regulated in various malignancies [20]. BIRC5 is also reported to be one of the prognostic markers for lung adenocarcinoma [21]. Moreover, BIRC5 up-regulation was previously documented in lung adenocarcinoma cells and tissues, wherein = radiosensitivity could affect the dependence on BIRC5 expression [11]. Consistently, RT-qPCR and Western blotting results in our study revealed that BIRC5 was highly-expressed in lung adenocarcinoma. Although the regulating relation between NPs and BIRC5 remains elusive, Survivin, another member of inhibitor of apoptosis protein family, is known to be knocked-down by NPs in MG-63 cells [22, 23]. Expanding on this, our findings displayed that Fe_3_O_4_ MNPs could reduce the BIRC5 expression in lung adenocarcinoma cells by delivering siBIRC5 and AS-ODN into lung adenocarcinoma cells simultaneously. On the other hand, the expression of DR5, a member of the tumor necrosis factor receptor superfamily (TNFRSF10B), was previously correlated with radiosensitivity in tumor cells [24]. Interestingly, the current study demonstrated that NPs could increase the DR5 expression by silencing BIRC5. NPs are further known to possess the ability to up-regulate DR5 to promote the apoptosis of colorectal cancer cells [25]. To our best knowledge, studies investigating the correlation between BIRC5 and DR5 remain scarce, therefore it would be plausible to suggest that BIRC5/DR5 may be a novel strategy involved in lung adenocarcinoma therapy. Furthermore, we also uncovered that co-treatment of NPs and radiotherapy brought about marked elevation in cell apoptosis of lung adenocarcinoma, as evidenced by up-regulated Caspase-3 expressions. Consistently, a prior study illustrated that RBCm-OM/PLGA nanoparticles promoted the apoptosis of non-small-cell lung adenocarcinoma cells by reducing the Bcl-2 expression and elevating Caspase-3 levels [26].

Additional in vivo experimentation in our study verified that NPs could enhance the radiosensitivity in vivo by down-regulating BIRC5 and up-regulating DR5, as witnessed by reduced tumor growth and down-regulated Ki67 positive expressions. The latter is particularly important as reduced Ki67 levels are correlated with enhanced radiosensitivity of lung adenocarcinoma after MG132 treatment [27]. Nevertheless, the side effects and toxicity of NPs remain a serious concern which limits its application in biomedicine [28]. Functional test including ALP and ALT are commonly used to detect the biocompatibility [29]. Our evaluation on biocompatibility suggested that the Fe_3_O_4_ MNPs were without obvious side effects for our in vivo models.

## Conclusion

To summarize, findings obtained in our study indicated that Fe_3_O_4_ MNPs could co-deliver siBIRC5 and AS-ODN to lung adenocarcinoma cells, which may be used as a potent radiosensitizer for treating lung adenocarcinoma in radiotherapy. Fe_3_O_4_ MNPs-targeted delivery of siBIRC5 and AS-ODN enhances radiosensitivity, which provides a novel therapeutic target for lung adenocarcinoma with a low risk of toxicity. Our prepared Fe_3_O_4_ MNPs loaded with AS-ODN and siBIRC5 provide a strong basis for enhancing the sensitivity of patients to radiotherapy. We will further investigate the tracer role Fe_3_O_4_ MNPs in vivo in our future endeavors, and also confirm the effect of Fe_3_O_4_ MNPs/siBIRC5/DR5 on radiotherapy. However, the novel function of Fe_3_O_4_ MNP-targeted siBIRC5 and AS-ODN might be a potential radiosensitizer for treating lung adenocarcinoma in the clinic. In order to accelerate the rapid conversion of Fe3O4 MNPs/siBIRC5/DR5 into clinical use, we will further study the metabolism mechanism of Fe3O4 MNPs/siBIRC5/DR5 in vivo and improve the safety data of internal circulation.

## Supplementary Information

**Additional file1:** **Figure S1.** NPs are successfully prepared. A, Schematic diagram of Fe3O4 MNPs preparation. B, FTIR spectra of intermediates at each step of Fe3O4 preparation. B1: Fe3O4 nuclei; B2: Fe3O4-PEG-LAC; B3: Fe3O4-PEG-LAC-chitosan; B4: Fe3O4-PEG-LAC-chitosan-PEI. C, Binding ability among Fe3O4 MNPs, AS-ODN, and siBIRC5 by electrophoresis (ratio in the Figure represents Fe3O4 MNPs : siBIRC5 : AS-ODN mass ratio). D, TEM images of NPs (scale bar: 100 nm). E, Size distribution of NPs in Figure A. F, SEM image of NPs (scale bar: 100 nm).

**Additional file2:** **Figure S2.** Survival rate of tumor cells at different concentrations of NPs and different radiation doses.

**Additional file3:** **Table S1.** Primer sequences used for RT-qPCR.

## Data Availability

The datasets generated during the current study are available.

## Abbreviations

NPs: Nanomaterials
MNPs: Magnetic nanoparticles
BIRC5: Baculoviral IAP repeat containing 5
AS-ODN: Antisense oligodeoxynucleotide
siRNA: Small interference RNA
DR5: Death receptor 5
siBIRC5: Small interfering RNA against BIRC5
PEG-LAC: Polyethylene glycol lactide
FTIR: Fourier transform infrared reflection
MEM: Minimum essential medium
RIPA: Radio immunoprecipitation
BCA: Bicinchoninic Acid
SDS: Sodium dodecyl sulfate
PAGE: Polyacrylamide gel electrophoresis
HRP: Horseradish peroxide
BSA: Bovine serum albumin
ELISA: Enzyme-linked immunoassay
DAB: Diaminobenzidine
ALT: Alanine aminotransferase
ALP: Alkaline phosphatase
AST: Aspartate aminotransferase
