# Supplementary figures and images for: Fe3O4 magnetic nanoparticle-enhanced radiotherapy for lung adenocarcinoma via delivery of siBIRC5 and AS-ODN
Source: J Transl Med. 2021 Aug 9;19:337. doi: 10.1186/s12967-021-02971-7 (PMC8351328; doi:10.1186/s12967-021-02971-7)

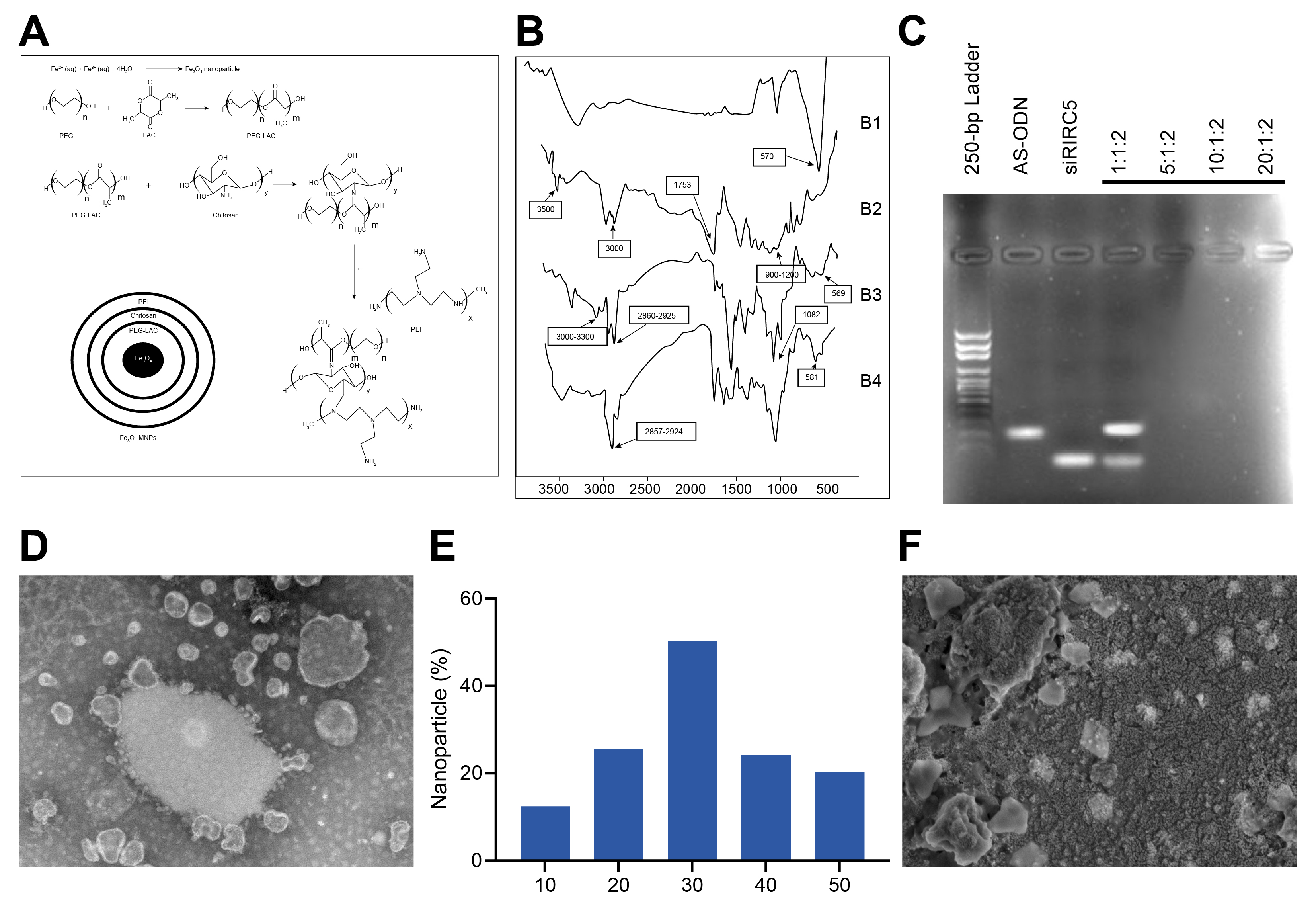

Supplement: Supplementary file 1 — Additional file1: Figure S1. NPs are successfully prepared. A, Schematic diagram of Fe3O4 MNPs preparation. B, FTIR spectra of intermediates at each step of Fe3O4 preparation. B1: Fe3O4 nuclei; B2: Fe3O4-PEG-LAC; B3: Fe3O4-PEG-LAC-chitosan; B4: Fe3O4-PEG-LAC-chitosan-PEI. C, Binding ability among Fe3O4 MNPs, AS-ODN, and siBIRC5 by electrophoresis (ratio in the Figure represents Fe3O4 MNPs : siBIRC5 : AS-ODN mass ratio). D, TEM images of NPs (scale bar: 100 nm). E, Size distribution of NPs in Figure A. F, SEM image of NPs (scale bar: 100 nm). [file 12967_2021_2971_MOESM1_ESM.jpg]

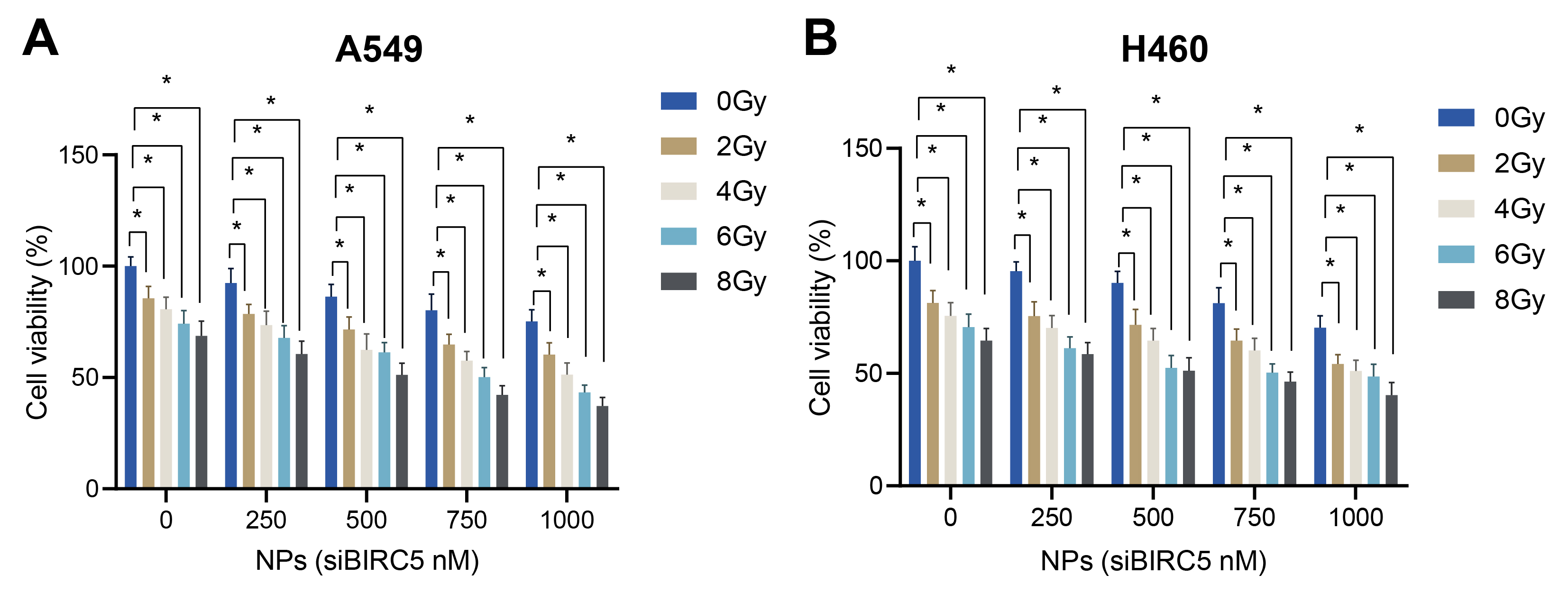

Supplement: Supplementary file 2 — Additional file2: Figure S2. Survival rate of tumor cells at different concentrations of NPs and different radiation doses. [file 12967_2021_2971_MOESM2_ESM.jpg]
