# Supplementary material for: Fe3O4 magnetic nanoparticle-enhanced radiotherapy for lung adenocarcinoma via delivery of siBIRC5 and AS-ODN
Source: J Transl Med. 2021 Aug 9;19:337. doi: 10.1186/s12967-021-02971-7 (PMC8351328; doi:10.1186/s12967-021-02971-7)
Supplement: Supplementary file 3 — Additional file3: Table S1. Primer sequences used for RT-qPCR. [file 12967_2021_2971_MOESM3_ESM.docx]

**Supplementary Table 1.** Primer sequences used for RT-qPCR.

|  | Forward (5’→3’) | Reverse (5’→3’) |
| --- | --- | --- |
| BIRC5 | CGAGGCTGGCTTCATCCA | CAACCGGACGAATGCTTTTT |
| DR5 | GACTCTGAGACAGTGCTTCGATGA | CCATGAGGCCCAACTTCCT |
| β-actin | CATCCGTAAAGACCTCTATGCCAAC | ATGGAGCCACCGATCCACA |

Notes: BIRC5, Baculoviral IAP repeat containing 5; DR5, Death Receptor 5
